# Koch vs. Virchow

**Published:** 1891-09

**Authors:** 


BUFFALO MEDICAL AND SURGICAL JOURNAL

A MONTHLY REVIEW OF MEDICINE AND SURGERY.

*EDITORS:*

THOMAS LOTHROP, M. D. - - WM. WARREN POTTER, M. D.

All communications, whether of a literary or business character, should be addressed to the managing editor : 284 FRANKLIN STREET, BUFFALO, N. Y.

VOL. XXXI. SEPTEMBER, 1891. No. 2.

### KOCH VS. VIRCHOW.

WE NOTICE, just at present, in many of our exchanges, a vein of jest, if not abuse, directed against the greatest of modern experimenters—Professor Koch, of Berlin. Ever since the publication of Virchow's manifesto, denouncing everything connected with the "lymph" question, the tirade of abuse began. Perhaps it will be well to bear in mind that Prof. Virchow was equally as denunciatory of Prof. Koch and his methods in 1882, when the latter, through his wonderful discovery of the bacillus, let the bottom fall out of Virchow's pet tuberculosis theory. Time has proven who was right, and we have no doubt but that in ten years from now, time will again demonstrate that all advancements do not emanate from Prof. Virchow.

In a recent debate in the lower house of the Prussian Diet, Prof. Virchow denounced Kochism as a failure, and opposed with all his power (?) the granting of 165,000 marks for Prof. Koch's Institute. Happily, Prof. Virchow is not as successful in politics as in science, and the honor of German medicine was sustained. We do not propose to belittle the magnitude and influence of Virchow's achievements, but when we see him at his old tricks, and this time transferrıng a scientific question into the political arena, we think he had better be in other business.

We still hold to the belief that Prof. Koch is on the right trail, and judging the man by his past efforts, we will be sadly mistaken if his experiments are not crowned with success. It requires time to work out such problems as "the cure of tuberculosis," but when pluck, brains and modesty are all combined in one head, we need not fear the result. Professor Koch deserves as much honor and appreciation now as he did before his adversaries took him to task on a point which was unwillingly wrenched from him.

